# Anti-Fibrotic Effects of Class I HDAC Inhibitor, Mocetinostat Is Associated with IL-6/Stat3 Signaling in Ischemic Heart Failure

**DOI:** 10.3390/ijms160511482

**Published:** 2015-05-19

**Authors:** Hikmet Nural-Guvener, Liudmila Zakharova, Lorraine Feehery, Snjezana Sljukic, Mohamed Gaballa

**Affiliations:** Cardiovascular Research Laboratory, Banner Sun Health Research Institute, Sun City, AZ 85351, USA; E-Mails: feyzaguvener@gmail.com (H.N.-G.); hbssmode@yahoo.com (L.Z.); lorraine.feehery@bannerhealth.com (L.F.); zana.sljukic@gmail.com (S.S.)

**Keywords:** congestive heart failure, myocardial infarction, Mocetinostat, cardiac fibrosis, HDAC

## Abstract

Background: Recent studies have linked histone deacetylases (HDAC) to remodeling of the heart and cardiac fibrosis in heart failure. However, the molecular mechanisms linking chromatin remodeling events with observed anti-fibrotic effects are unknown. Here, we investigated the molecular players involved in anti-fibrotic effects of HDAC inhibition in congestive heart failure (CHF) myocardium and cardiac fibroblasts *in vivo*. Methods and Results: MI was created by coronary artery occlusion. Class I HDACs were inhibited in three-week post MI rats by intraperitoneal injection of Mocetinostat (20 mg/kg/day) for duration of three weeks. Cardiac function and heart tissue were analyzed at six week post-MI. CD90^+^ cardiac fibroblasts were isolated from ventricles through enzymatic digestion of heart. *In vivo* treatment of CHF animals with Mocetinostat reduced CHF-dependent up-regulation of HDAC1 and HDAC2 in CHF myocardium, improved cardiac function and decreased scar size and total collagen amount. Moreover, expression of pro-fibrotic markers, collagen-1, fibronectin and Connective Tissue Growth Factor (CTGF) were reduced in the left ventricle (LV) of Mocetinostat-treated CHF hearts. Cardiac fibroblasts isolated from Mocetinostat-treated CHF ventricles showed a decrease in expression of collagen I and III, fibronectin and Timp1. In addition, Mocetinostat attenuated CHF-induced elevation of IL-6 levels in CHF myocardium and cardiac fibroblasts. In parallel, levels of pSTAT3 were reduced via Mocetinostat in CHF myocardium. Conclusions: Anti-fibrotic effects of Mocetinostat in CHF are associated with the IL-6/STAT3 signaling pathway. In addition, our study demonstrates *in vivo* regulation of cardiac fibroblasts via HDAC inhibition.

## 1. Introduction

After myocardial infarction (MI), there are several adaptive and maladaptive processes that occur in the heart leading to heart failure. These processes include the loss of cardiomyocytes, scar formation and deterioration of contractile function. The CHF environment is associated with interstitial fibrosis; transformation of cardiac fibroblasts into an active fibroblast phenotype (myofibroblasts) plays a critical role in this process [1,2,3]. While myofibroblasts serve an important role in adaptive responses such as wound healing and scar formation, further deposition of interstitial ECM causes myocardial stiffness and loss of ventricular function. Therefore, dissecting the molecular mechanisms leading to interstitial fibrosis and development of anti-fibrotic strategies to combat heart failure is of great interest.

Recent studies identified epigenetic modifications of chromatin as a new tool to regulate cardiac physiology. Thus, activity of the histone deacetylase (HDAC) family, enzymes that catalyze the removal of acetyl groups from proteins, were linked with heart failure associated remodeling including development and progression of cardiac fibrosis [4,5,6,7,8,9,10]. To date, eighteen mammalian HDACs were identified and categorized into four classes. Class I HDACs (HDAC1, 2, 3 and 8) are widely expressed and have a pro-hypertrophic function in heart disease. HDAC inhibitors, especially Class I and II inhibitors, were shown to decrease interstitial fibrosis, and improve cardiac function in pathological heart conditions [6,10,11,12,13]. We recently showed that inhibition of class I HDACs with Mocetinostat reduced cardiac fibrosis in CHF hearts and diminished cardiac myofibroblast activation [8]. However, the mechanisms underlying the anti-fibrotic effects of HDAC inhibition remains unclear.

A number of cytokines and growth factors are linked with development of cardiac fibrosis [14,15]. Of these molecules, CTGF and IL-6 are considered among the markers of heart failure [15,16,17]. The level of circulating IL-6 is increased in heart failure patients correlating with severity of the disease [18,19]. In addition, studies in rats with post-infarction heart failure indicates that mRNA levels of IL-6 are elevated in non-infarcted myocardium [20]. In a recent study, inhibition of class 1 and 2 HDACs resulted in reduction of circulating levels of several pro-inflammatory cytokines including IL-6 in rats with induced hypertensive cardiomyopathy [4]. However, it is unclear whether anti-fibrotic effects of HDAC inhibition in CHF are associated with modulation of pro-inflammatory cytokines. Therefore, we investigated the effects of systemic HDAC inhibition on several cytokines including CTGF and IL-6 in CHF animals.

The IL-6 family of cytokines mediates their actions mainly through Janus kinase signal transducers and activators of transcription (JAK/STAT) pathways in addition to other signaling pathways [21]. There are seven STAT proteins that have been identified so far which are all expressed in the heart. IL-6 is a prominent activator of STAT3 which plays a central role in cardiac physiology and pathophysiology [22]. Although there are conflicting results about the role of STAT3 activation being whether it is beneficial or detrimental in response to MI, recent evidence indicates that the sustained activation of STAT3 signaling after MI may contribute to adverse remodeling and progression to heart failure [23]. Increasing evidence suggests a role for HDACs in STAT activation. In a recent study, HDAC-mediated activation of renal fibroblasts was involved in the activation of STAT3 signaling [24]. In another study, HDAC inhibition was followed by a decrease in STAT3 in a model of experimental colitis [25]. Here, we investigated whether anti-fibrotic effects of HDAC inhibition modulate STAT3 signaling in CHF myocardium.

In the present study, we showed that Mocetinostat at 20 mg/kg/day reduced HDAC1 and 2 levels in CHF myocardium. In addition, Mocetinostat improved cardiac function and reduced interstitial fibrosis and scar size in CHF. Moreover, Mocetinostat reduced the expression of pro-fibrotic cytokine CTGF as well as ECM components in CHF myocardium. Interestingly, Mocetinostat attenuated CHF induced up-regulation of IL-6 in myocardium and cardiac fibroblasts and reduced activation of STAT3 proteins in the myocardium. In parallel, expression of ECM components and their regulators were reduced in cardiac fibroblasts isolated from Mocetinostat treated CHF hearts. Thus, the anti-fibrotic activity of Mocetinostat includes modulation of IL-6/STAT3 axis and down-regulation of ECM production in myocardium and cardiac fibroblasts *in vivo.*

## 2. Results

Previously, we and others showed the beneficial effects of Class I HDAC inhibitor Mocetinostat at the dose of 10 mg/kg/day on cardiac function and fibrosis in cardiac disease [8,9]. However, we observed only partial improvement in cardiac function with no change in scar size in CHF after treatment with Mocetinostat at 10 mg/kg/day. Here, we investigated whether beneficial effects of HDAC inhibition on CHF rats would further increase with a new dose of Mocetinostat (20 mg/kg/day). CHF animals were treated with vehicle or 20 mg/kg/day (ip) Mocetinostat starting three weeks after MI for duration of three weeks. Cardiac function and heart tissue were analyzed at six weeks post-MI.

### 2.1. Mocetinostat Reduced Levels of HDAC1 and HDAC2 in CHF

**Figure 1:**
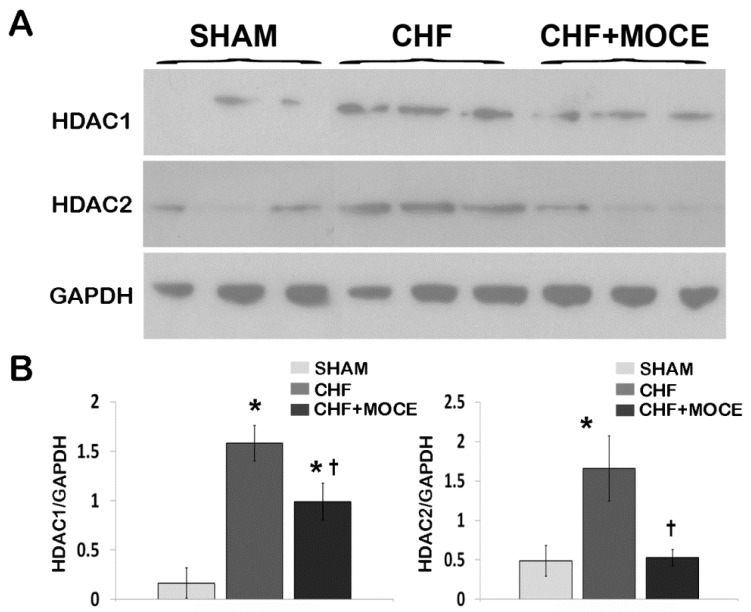
Mocetinostat reduced HDAC1 and 2 levels in CHF. (**A**) LV from sham, untreated and Mocetinostat-treated CHF hearts was lysed. Forty µg of protein was loaded and separated in 4%–12% Bis-Tris SDS/PAGE gel and blotted with HDAC1 and HDAC2 antibodies. GAPDH was used to normalize for loading (*n* = 4); (**B**) Density analysis of blots was presented as arbitrary units in bar graphs. Error bars indicate SE. *, *p* < 0.05 compared to shams; †, *p* < 0.05 compared to untreated CHF; CHF, congestive heart failure; HDAC, Histone Deacetylase; MOCE, Mocetinostat.

Previously, we showed that HDAC1 and 2 were up-regulated in CHF myocardium [8]. Therefore, we investigated whether class I HDAC inhibition with Mocetinostat attenuate up-regulation of HDAC1 and 2 in the CHF myocardium. Western blot analysis was performed with the tissue isolated from left ventricles (LV) (non-scar tissue) of sham; Mocetinostat treated and untreated CHF rats (Figure 1). HDAC1 and 2 levels were up-regulated in LV of untreated CHF animals compared to sham. On the other hand, HDAC1 and 2 levels were reduced in the LV of Mocetinostat-treated CHF rats in comparison to untreated CHF rats. Thus, Mocetinostat reduced the CHF-dependent elevation of HDAC1 and 2 levels in LV.

### 2.2. Mocetinostat at 20 mg/kg Improved Cardiac Function in CHF

**Figure 2:**
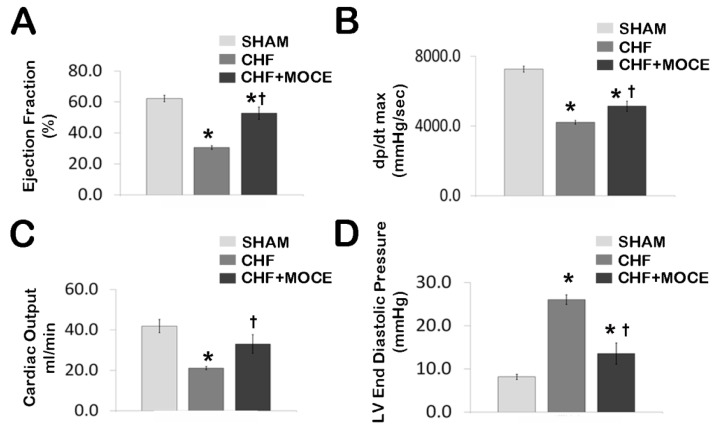
Mocetinostat improved cardiac function in CHF. Hemodynamic parameters were measured in sham, untreated and Mocetinostat (20 mg/kg)-treated CHF and presented in bar graphs; (**A**) Ejection Fraction (**B**) dp/dt max (**C**) Cardiac output (**D**) Left ventricle end diastolic pressure. Sham (*n* = 10), CHF (*n* = 10), CHF + MOCE (*n* = 10). Error bars indicate SE. *, *p* < 0.05 compared to sham; †, *p* < 0.05 compared to CHF; CHF, congestive heart failure; LV, left ventricle; dP/dt_max_, peak rate of left ventricular pressure rise; MOCE, Mocetinostat.

Cardiac function was measured in closed-chest pressure-volume loops generated using a solid-state Millar conductance catheter system in sham, Mocetinostat-treated and untreated CHF animals. Untreated CHF animals had a significant loss of cardiac function compared to sham animals. When compared to shams, untreated CHF rats left ventricle ejection fraction was decreased from 62.2% ± 2.1% to 30.5% ± 1.2%, dP/dt_max_ was decreased to 4201 ± 104 from 7253 ± 174 mmHg/s and cardiac output was decreased from 41.8 ± 3.2 to 21.1 ± 0.8 mL/min, while left ventricular end-diastolic pressure (LVEDP) was increased from 8.2 ± 0.5 to 26 ± 1 mmHg, (Figure 2), confirming heart failure [26]. Twenty mg/kg/day of Mocetinostat administration in CHF animals resulted in improvement of left ventricular contractility markers; ejection fraction increased to 52.8% ± 4% (Figure 2A) and dP/dt_max_ to 5128 ± 293 mmHg/s (Figure 2B) as well as cardiac output to 32.9 ± 4.7 mL/min (Figure 2C) compared to untreated CHF animals. In addition, LVEDP (Figure 2D) was significantly reduced to 13.6 ± 2.3 mmHg in Mocetinostat treated animals compared to untreated CHF rats. Thus, Mocetinostat administration at 20 mg/kg/day improved left ventricle contractility markers in CHF hearts.

### 2.3. Mocetinostat at 20 mg/kg/day Reduced Interstitial Fibrosis and Scar Size

**Figure 3:**
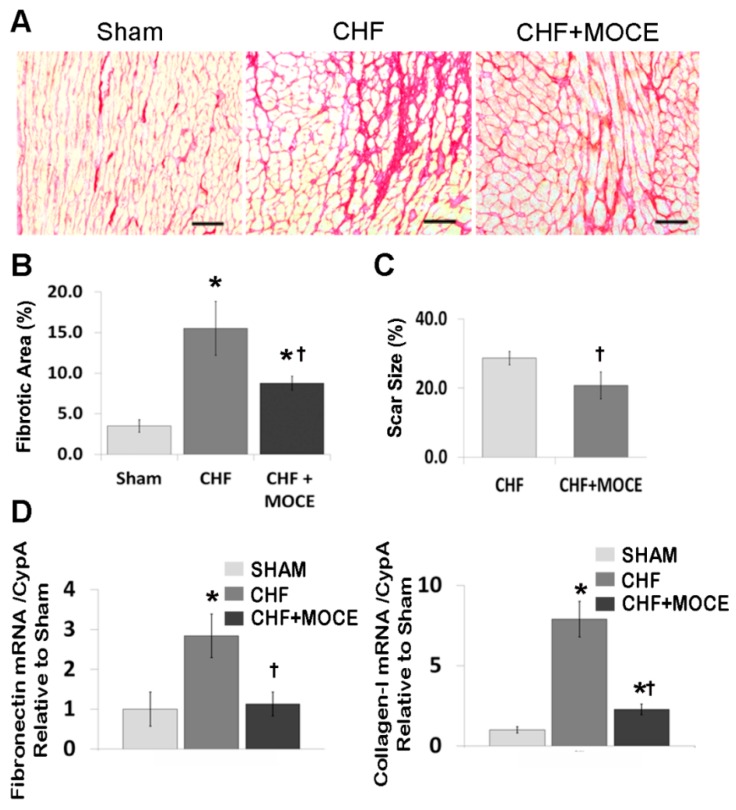
Mocetinostat reduced fibrosis and scar size. (**A**) Total collagen in heart sections were stained with Sirus Red in untreated and Mocetinostat-treated CHF animals. Representative images are shown. Scale bar, 50 µm; (**B**) Total collagen amount was quantified in Sirus red stained sections; (**C**) Scar size was measured in Trichrome-stained heart sections of untreated or Mocetinostat-treated CHF rats; (**D**) QPCR analysis was performed to measure mRNA levels of fibronectin and collagen-I in LV of sham, untreated and Mocetinostat treated CHF animals. Error bars indicate SE. MOCE, Mocetinostat; CHF, congestive heart failure; *, *p* < 0.05 compared to shams; †, *p* < 0.05 compared to untreated CHF.

Next, we measured total collagen amount with Sirius red staining in cross sections of sham and CHF hearts treated with Mocetinostat (20 mg/kg/day) or vehicle (Figure 3A). In sham hearts, total collagen amount was 3.5% ± 0.8%, while its percentage increased to 15.5% ± 3.3% in CHF animals. Mocetinostat treatment reduced the total collagen amount to 8.8% ± 0.9% (Figure 3B). In addition, trichrome staining was performed to assess scar size. Mocetinostat reduced the scar size to 20.8% from 28.7% in CHF hearts (Figure 3C). Moreover, we measured the mRNA levels of ECM components fibronectin and collagen-1 in left ventricular tissue. In untreated CHF rats, expression of both genes was increased compared to sham, while Mocetinostat treatment significantly attenuated the CHF-induced increase in fibronectin and collagen-1 levels (Figure 3D). Thus, Mocetinostat at 20 mg/kg/day effectively reduced interstitial fibrosis and scar size.

One of the effects of HDAC inhibitors is their ability to induce cell cycle arrest and apoptosis [27]. To test whether Mocetinostat at the dose of 20 mg/kg/day induce apoptosis in CHF myocardium, we measured the number of positive cleaved-caspase-3 cells in the scar and LV of Mocetinostat-treated and untreated CHF animals (Supplementary Figure S1). There were no differences in the number of cleaved-caspase-3 positive cells within the scar region and LV of Mocetinostat treated versus untreated CHF animals. Therefore, Mocetinostat at 20 mg/kg/day did not affect the rate of apoptosis neither in LV nor in the scar region of CHF animals.

### 2.4. Mocetinostat Reduced the Expression of Connective Tissue Growth Factor in CHF Myocardium

**Figure 4:**
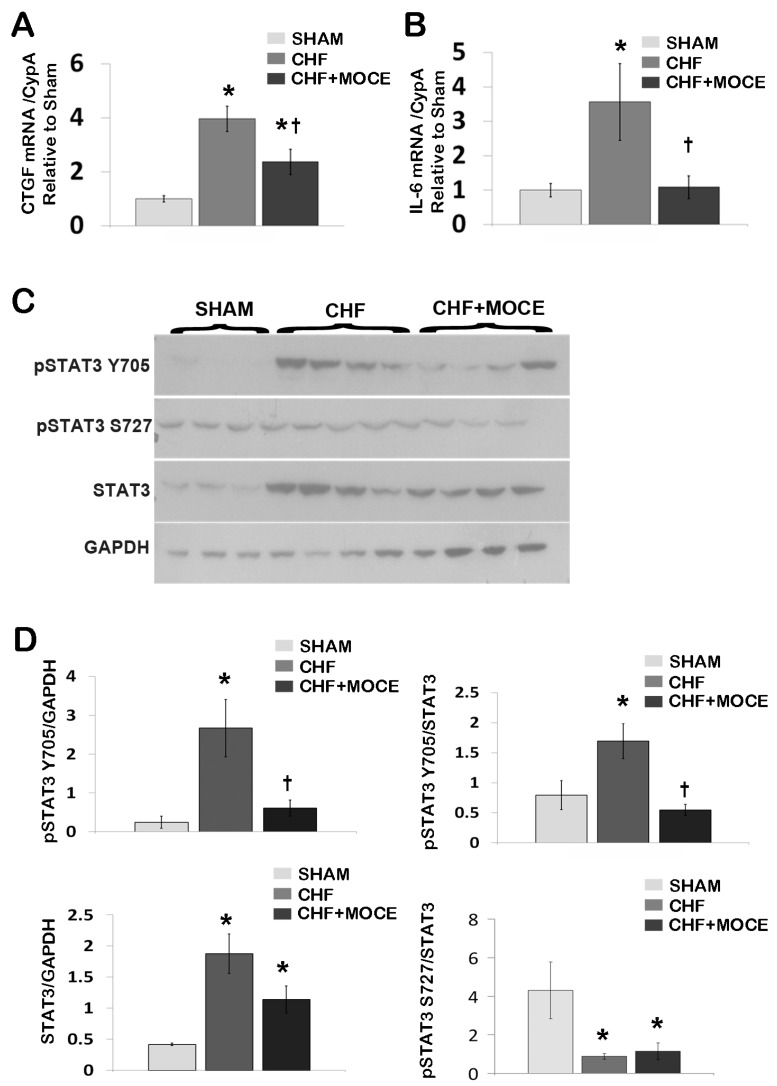
Mocetinostat reduced IL-6 and p-Stat3 levels in CHF. Gene expression of CTGF (**A**) and IL-6 (**B**) in LV of sham, untreated and Mocetinostat-treated CHF hearts were analyzed with real-time PCR (*n* = 5); (**C**) LV from sham, untreated and Mocetinostat-treated CHF hearts were lysed and separated in 4%–12% Bis-Tris SDS/PAGE gel and blotted with pSTAT3 (Y705 and S727) and STAT3 antibodies. GAPDH was used to normalize for loading. (*n* = 4); (**D**) Density analysis of blots was presented as arbitrary units in bar graphs. Error bars indicate SE. *, *p* < 0.05 compared to shams; †, *p* < 0.05 compared to untreated CHF; CHF, congestive heart failure; CTGF, connective tissue growth factor; IL-6, interleukin-6; MOCE, Mocetinostat.

In our current study, we showed that Mocetinostat reduced interstitial fibrosis in CHF animals; however the key fibrotic pathways that are affected by Mocetinostat are unknown. Here, we investigated the effects of Mocetinostat on the expression of fibrogenic cytokines; transforming growth factor β (TGF-β), connective tissue growth factor (CTGF) and platelet derived growth factor (PDGF). We did not observe any significant changes in expression of TGF-β or PDGF in the LV of Mocetinostat treated or untreated CHF compared to sham rats (data not shown). In contrast, CTGF expression was increased in the LV of untreated CHF in comparison to sham rats (3.96-fold, Figure 4A), while Mocetinostat treatment significantly reduced the expression level of CTGF. CTGF, which is a down-stream signaling component of TGF-β signaling pathway, is considered an important pro-fibrotic growth factor and a marker of heart failure. Thus, modulation of CTGF is associated with anti-fibrotic effects of Mocetinostat in CHF.

### 2.5. Mocetinostat Modulates IL-6 and Stat3 Signaling in CHF Myocardium

Next, we investigated the effects of Mocetinostat on inflammatory cytokines such as IL-6 and IL-18. IL-6 expression was increased in the LV of untreated CHF compared to sham (3.6-fold, Figure 4B). Mocetinostat treatment lowered the level of IL-6 back to the basal levels in CHF LV. On the other hand, there were no significant changes in IL-18 levels in the LV of Mocetinostat-treated or untreated CHF compared to sham rats.

The IL-6 family of cytokines mediates their action primarily through the activation of Janus kinase signal transducers and activators of the transcription (JAK-STAT) pathway [28]. Therefore, we investigated the levels of STAT3 and phosphorylated STAT3 at two residues Y705 and S727 in the LV of Mocetinostat-treated or untreated CHF and sham hearts. Western blot analysis revealed that the level of STAT3 was increased in untreated CHF (4.5-fold) compared to sham (Figure 4C,D). Mocetinostat showed a decreasing trend in the level of STAT3 protein in LV of treated CHF compared to untreated CHF animals (*p* = 0.15). In addition, the level of pSTAT3-Y705 and the ratio of pSTAT3 Y705/STAT3 were increased in untreated CHF LV compared to sham hearts (10.8- and 2.1-fold, respectively; Figure 4C,D). Meanwhile, Mocetinostat treatment reduced the level of pSTAT3 Y705 and the ratio of pSTAT3 Y705/STAT3 in LV of CHF hearts compared to untreated CHF hearts. In contrast, we did not observe any changes in the level of pSTAT3 S727 in the LV of Mocetinostat treated or untreated CHF compared to sham animals. However, the ratio of pSTAT3 Y272 /STAT3 was reduced in LV of Mocetinostat treated or untreated CHF compared to sham hearts (Figure 4C,D). Thus, Mocetinostat treatment attenuated the CHF-induced activation of STAT3 in LV.

### 2.6. Mocetinostat Reduced Fibronectin and Collagen Levels in Cardiac Fibroblasts

**Figure 5:**
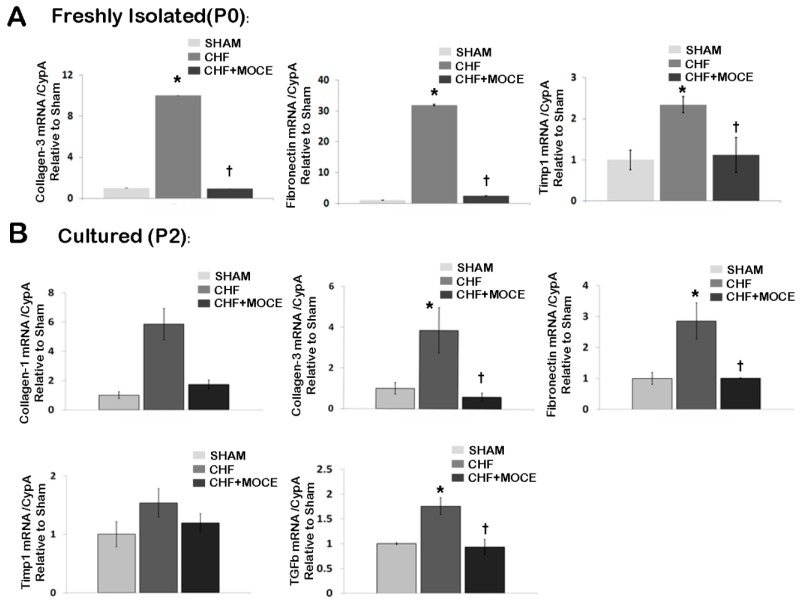
Mocetinostat reduced expression of ECM-related genes in endogenous cardiac fibroblasts. (**A**) Gene expression of collagen-3, fibronectin, Timp1 were measured with real-time PCR in CD90^+^ cardiac fibroblasts isolated from untreated and Mocetinostat-treated CHF hearts at P0 without culturing in *in vitro*; (**B**) Gene expressions of collagen-3, fibronectin, and TGFβ were measured with real-time PCR in cultured (P2) CD90^+^ cardiac fibroblasts isolated from untreated and Mocetinostat-treated CHF hearts. Error bars indicate SE. (*n* = 5); *, *p* < 0.05 compared to shams; †, *p* < 0.05 compared to untreated CHF; CHF, congestive heart failure; MOCE, Mocetinostat.

Recently, we and others showed that HDAC inhibition via Mocetinostat modulated cardiac fibroblast activation and proliferation *in vitro* [8,9]. Here, we investigated the effects of Mocetinostat on cardiac fibroblasts in CHF *in vivo*. We treated CHF rats with Mocetinostat or saline then digested the hearts to isolate CD90^+^ cardiac fibroblasts. First, we assessed expressions of ECM-related genes in freshly isolated fibroblasts (P0) (Figure 5A). We observed an increase in the expression levels of collagen-III, fibronectin and Tissue inhibitors of metalloproteinase-1 (Timp1) in fibroblasts isolated from untreated CHF ventricles compared to sham. Interestingly, in cardiac fibroblasts isolated from Mocetinostat-treated CHF ventricles, expression levels of collagen-III, fibronectin and Timp1 were decreased when compared to their counterparts isolated from untreated CHF ventricles. Thus, Mocetinostat reduced expression of genes involved in ECM composition and regulation in fibroblasts *in vivo*.

Next, we investigated whether the effects of *in vivo* Mocetinostat treatment were stable in culture conditions, we cultured fibroblasts isolated from sham, Mocetinostat-treated and untreated CHF ventricles until passage 2 (P2). Then, we assessed gene expression levels of collagen-I and III, fibronectin, Timp1 and TGFβ (Figure 5B). Similar to freshly isolated fibroblasts, untreated CHF-derived fibroblasts at P2 also showed increases in collagen III and fibronectin levels. Moreover, we observed up-regulation of collagen I and TGFβ levels in cultured fibroblasts derived from untreated CHF ventricles compared to sham fibroblasts. In contrast, there were no changes in Timp1 level. On the other hand, fibroblasts derived from Mocetinostat-treated CHF ventricles had lower mRNA levels of collagen-I and III, fibronectin and TGFβ compared to their counterparts derived from untreated CHF ventricles. Thus, the effects of *in vivo* Mocetinostat treatment are stable in cultured fibroblasts, at least until P2.

### 2.7. Mocetinostat Reduced IL-6 Levels in Cardiac Fibroblast

**Figure 6:**
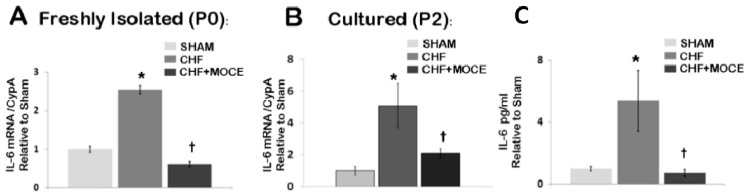
Mocetinostat reduced IL-6 level in cardiac fibroblasts. (**A**) Levels of IL-6 mRNA were measured in CD90^+^ fibroblasts isolated from sham, untreated and Mocetinostat treated CHF ventricles directly after isolation without culturing (P0) and (**B**) at P2 after culture; (**C**) ELISA was performed to measure levels of IL-6 in conditioned media of CD90^+^ fibroblasts isolated from sham, untreated and Mocetinostat treated CHF ventricles. Error bars indicate SE. (*n* = 5); *, *p* < 0.05 compared to shams; †, *p* < 0.05 compared to untreated CHF; CHF, congestive heart failure; MOCE, Mocetinostat.

Here, we showed that Mocetinostat reduced IL-6 levels in CHF myocardium. Cardiac fibroblasts have been shown to express IL-6 upon pathological stimuli [29,30]; therefore we assessed the level of IL-6 in cardiac fibroblasts isolated from ventricles of sham, CHF and Mocetinostat-treated CHF animals (Figure 6). In both freshly isolated and cultured fibroblasts derived from untreated CHF ventricles, IL-6 levels were up-regulated in comparison to sham derived fibroblasts (Figure 6A,B). In addition, levels of IL-6 protein were increased in conditioned media of cultured fibroblasts derived from untreated CHF ventricles (Figure 6C). Both mRNA and protein levels of IL-6 were significantly reduced in cardiac fibroblasts derived from Mocetinostat-treated CHF ventricles compared to their untreated CHF counterparts. Thus, *in vivo* Mocetinostat treatment in CHF animals reduced expression and protein levels of IL-6 in cardiac fibroblasts.

## 3. Discussion

Recent studies established a link between HDAC activity and cardiac fibrosis in various cardiac diseases including CHF. However, the molecular players involved in the anti-fibrotic effects of HDAC inhibition remain unknown. In this study, we investigated the effects of class I HDAC inhibition on CHF myocardium and cardiac fibroblasts *in vivo*. Class I HDAC inhibitor, Mocetinostat attenuated CHF induced up-regulation of HDAC1 and HDAC2 in LV. Twenty mg/kg/day of Mocetinostat administration improved cardiac function, reduced interstitial fibrosis and scar size in CHF. Mocetinostat attenuated CHF-induced up-regulation of IL-6 in myocardium and cardiac fibroblasts and reduced activation of STAT3 proteins in the myocardium. In parallel, expression of ECM components was reduced in cardiac fibroblasts isolated from Mocetinostat-treated CHF hearts. Thus, anti-fibrotic activity of Mocetinostat includes *in vivo* modulation of the IL-6/STAT3 axis and down-regulation of ECM production in myocardium and cardiac fibroblasts.

In this current study, we observed that Mocetinostat attenuated CHF-induced up-regulation of IL-6 levels in LV and in cardiac fibroblasts. Recent studies point toward regulatory effects of IL-6 in pathological fibrosis where IL-6 infusion in rats caused LV hypertrophy and myocardial fibrosis [31]. Thus, the anti-fibrotic actions of HDAC inhibition could be linked with down-regulation of IL-6 in the myocardium and cardiac fibroblasts. Suppression of IL-6 in cardiac fibroblasts through HDAC inhibition could be also associated with ECM production and cardiac fibrosis. In a recent study, it was shown that IL-6 is also involved in increased collagen synthesis in cardiac fibroblasts and heart [32]. In another study, IL-6 production in macrophage stimulated cardiac fibroblast resulted in activation of Tumor Growth Factor (TGF-β) signaling in fibroblasts which in turn stimulated cardiac fibrosis [29]. In line with current data, up-regulation of IL-6 in CHF myocardium and cardiac fibroblasts suggest a link between IL-6 and cardiac fibrosis in heart failure secondary to myocardial infarction. Therefore, IL-6 could be one of the molecular players involved in HDAC-mediated cardiac fibrosis.

Downstream signaling of IL-6 involves activation of the JAK-STAT pathway. In this study, we observed up-regulation of both total STAT-3 and phosphorylated STAT3 at Y705 proteins in LV of CHF while Mocetinostat treatment reduced activation of STAT3 in the CHF myocardium. In contrast, level of phosphorylated STAT3 at S727 was reduced in untreated and Mocetinostat treated CHF. Phosphorylation of STAT3 at Y705 is required for STAT3 activation, where phosphorylation of S727 regulated the transcriptional activity of STAT3. Recent studies suggest that STAT3 serine phosphorylation negatively modulates its tyrosine phosphorylation. Phosphorylation of S727 of STAT3 promotes dephosphorylation of STAT3 pY705 resulting in decrease in the duration of transcriptional activity. In glioma cells, reduced S727 phosphorylation of Stat3 with concomitant increase in Y705 phosphorylation leads to enhanced rate of proliferation and invasive property [33]. However, further studies need to be done to establish the role of phosphorylated STAT-3 at S727 in cardiac pathophysiology. There is a fine balance in the effects of IL-6/STAT3 signaling, since STAT3 activity in acute ischemia is cardioprotective, while continuous activation could be detrimental. In the heart, STAT3 activation has been shown to be cardioprotective, inducing angiogenesis and cell protection [34,35]. On the other hand, a recent study suggested that sustained activation of STAT3 signaling after MI may actually contribute to adverse remodeling and heart failure [23]. Elevated levels of phosphorylated STAT3 at Y705 were reported in failing human hearts [36]. In various disease models, inhibition of STAT3 resulted in down-regulation of collagen synthesis and fibrosis [28]. Here, we demonstrated activation of STAT3 in heart failure developed post MI. In these settings, an increase in STAT3 activation could have detrimental effects such as induction of collagen synthesis contributing to cardiac fibrosis. Inhibition of class I HDACs via Mocetinostat reduced the levels of phosphorylated STAT3 (Y705). Recently, in an obstructive neuropathy model, HDAC-mediated activation of renal fibroblasts involved activation of the STAT3 signaling pathway. Inhibition of HDAC activity with TSA attenuated STAT3 activation in renal fibroblasts [24]. Altogether, our results are in agreement with current studies, suggesting a possible link between HDAC activity and STAT3 activation in cardiac fibrosis in CHF. However, further studies are needed to delineate how HDAC inhibition regulates STAT3 activation in CHF myocardium.

In cardiac fibrosis increased amounts of collagen and other ECM proteins are deposited in the interstitium of the myocardium. Cardiac fibroblasts are the major producers of ECM during fibrotic processes. Here, we showed that systemic Mocetinostat administration to CHF animals reduced the expression of ECM components collagen and fibronectin and their regulators such as Timp-1 in cardiac fibroblasts *in vivo*. *In vitro* experiments showed that HDAC inhibitors regulated cardiac fibroblast proliferation, migration and activation [5,9]. In addition, in our previous study, we showed that Mocetinostat down-regulated the expression ECM components *in vitro* [8]. The majority of data on effects of HDAC inhibition on cardiac fibroblasts has been generated from *in vitro* cell culture models, while these current findings shed light on modulation of cardiac fibroblast biology *in vivo*. Moreover, we observed a similar pattern of gene expression profile in cultured cardiac fibroblasts derived from Mocetinostat treated CHF animals, indicating long-lasting effects of Mocetinostat on cardiac fibroblasts. One limitation of the study was that we isolated cardiac fibroblast from ventricular tissue based on the expression of the CD90 (Thy1) surface marker. CD90^+^ cells do not represent the entire fibroblast population in the heart; this marker is instead expressed on a subset of fibroblasts.

Previously, we showed that intra-peritoneal (ip) administration of 10 mg/kg/day of Mocetinostat in CHF improved left ventricle end diastolic pressure (LVEDP) and maximum rate of pressure rise (dP/dt_max_), with no effect on ejection fraction and cardiac output [8]. In study, we increased the dosage of Mocetinostat to 20 mg/kg/day to investigate whether higher dosage further improves cardiac function. At 20 mg/day/day Mocetinostat improved parameters of cardiac contractility including LVEDP, dP/dt_max_, ejection fraction and cardiac output. Administration of 10 and 20 mg/kg/day of Mocetinostat reduced fibrosis, but only 20 mg/kg/day reduced the scar size. Neither dose showed an increase in the apoptosis rate in the myocardium. Thus, Mocetinostat at 20 mg/kg/day is more effective in improving cardiac function and scar size.

Taken together, our results demonstrate that Mocetinostat modulates pro-fibrotic genes in cardiac fibroblasts and CHF myocardium *in vivo*. Specifically, the IL-6/STAT3 signaling pathway could be associated with the anti-fibrotic effects of Mocetinostat in CHF. This knowledge is important to further understand specific targets of HDAC inhibition in heart failure and develop therapeutic agents to reduce fibrosis.

## 4. Experimental Section

This study was performed in a facility accredited by the American Association for Accreditation of Laboratory Animal Care and was approved by the Institutional Animal Care and Use Committee at Banner Sun Health Research Institute. Animals received humane care in compliance with the Guide for the Care and Use of Laboratory Animals published by the US National Institutes of Health (NIH Publication No. 85-23, revised 1996).

### 4.1. Myocardial Infarction and Treatments

MI was created by ligation of the left coronary artery as previously performed by our laboratory [37]. In brief, rats were anesthetized using 1 mL/kg of an MI cocktail composed of ketamine (50 mg/mL), xylazine (15 mg/mL), acepromazine (2 mg/mL), and atropine (1 mg/mL). Animals were intubated and ventilated using a small animal ventilator (Harvard Apparatus, Holliston, MA, USA). A left thoracotomy was performed via the third intercostal rib, and the left coronary artery was ligated. In sham-operated animals, the chest was closed without ligation of the artery. The rats were sacrificed at 6 weeks (Congestive Heart Failure-CHF) post-surgery. At 6 weeks after successful infarction, rats exhibited CHF indicated by elevation of left ventricular end-diastolic pressure (LVEDP), LV remodeling, and fluid accumulation in the chest [37,38]. In addition, prominent fibrosis in the ventricles and left atrium was evident at 6 weeks post-surgery. Closed-chest *in vivo* cardiac function was measured using a Millar pressure conductance catheter system (Millar instruments, Houston, TX, USA) as previously described [26].

At 3 weeks post-surgery, animals were randomly assigned to Mocetinostat- or saline-treated groups. The first group received 20 mg/kg/day Mocetinostat dissolved in 0.1 N HCl PBS solution daily for the duration of 3 weeks (*n* = 8). The second group received only vehicle for the same duration (*n* = 10). In addition, animals that underwent sham surgery were injected with vehicle only and served as control group (*n* = 5).

### 4.2. Cell Isolation and Culture

CD90 cells from ventricles were isolated as described earlier [8]. Briefly, ventricular tissue was cut into 1–2 mm^3^ pieces and digested with a Dispase II (2.4 mg/mL, Roche, Indianapolis, IN, USA)/Collagenase II (0.05 mg/mL, Gibco, Grand Island, NY, USA) mix in PBS for 10 min at 37 °C with agitation. The tissue suspension was vortexed for 10 s and the supernatant spun at 1200× *g* for 7 min to collect dissociated cells. The last two steps were repeated 5 times and cells were pooled. Dissociated cells were plated in DMEM/F12 (Gibco) supplemented with 10% fetal bovine serum (FBS, Lonza, Basel, Switzerland), 100 U/mL penicillin G, and 100 µg/mL streptomycin for 2 h. Non-attached cells and debris were discarded while attached cells were trypsinized for CD90 isolation. CD90^+^ cells were separated from the cell outgrowths using magnetic beads MACS (Miltenyi Biotec, San Diego, CA, USA) according to manufacturer protocol and analyzed by flow cytometry for purity assessment.

### 4.3. Immunostaining

Coronal and axial tissue sections (5–7 μm thickness) were mounted on positively charged glass slides. Sections were fixed with 3% formaldehyde in PBS for 10 min at room temperature and were permeabilized in 100% methanol. Sections were blocked with 3% BSA in PBS and incubated with primary antibody against cleaved-caspase-3 (Cell Signaling Technologies, Beverly, MA, USA). Specific staining was visualized using corresponding secondary antibodies conjugated with Alexa 488 or 568 (Molecular Probes, Omaha, NE, USA). Nuclei were stained with DAPI, 4',6-diamidino-2-phenylindole (Life Technologies, Grand Island, NY, USA). For quantification, 5 images for region at 10× magnifications were captured using Leica TCS SPE confocal system (Buffalo Grove, IL, USA) configured with Leica DM 2500 microscope. The number of positive cells was quantified with Image J software (NIH, National Institute of Health, Bethesda, MD, USA).

### 4.4. Scar Size Assessment and Collagen Assay

Heart tissue was embedded in tissue freezing media (Sakura Tissue-Tek, Torrance, MA, USA) snap-frozen in liquid nitrogen and sectioned in the axial plane using Leica CM1900 cryostat (Leica Microsystems, Bannockburn, IL, USA). Axial tissue sections (5–7 μm thickness) were mounted on positively charged glass slides. For scar size assessment, sections were stained with Masson’s Trichrome kit (Sigma-Aldrich, St. Louis, MO, USA) according to the manufacturer’s protocol. Transmitted light images of heart sections were processed using DP2-BSW software (Olympus Corp, Phoenix, AZ, USA). Scar percentage was calculated as a ratio of collagen enriched scar area (blue staining) to the whole left ventricle area (red staining).

Total collagen amount was measured in heart section stained with Picrosirius red. Briefly, air-dried 5 µm heart sections were immersed in xylene for 10 min. The sections were treated with ethanol through descending concentrations of 100%, 95%, and 70% and rinsed in distilled water. The staining was performed in 0.1% Picrosirius red (Sigma Aldrich, St. Louis, MO, USA) solution for 7 min, followed with 2 min 0.01% N HCl rinse. Sections were dehydrated in ascending concentrations of ethanol and cleared in xylene before cover slipped with Permount (Fisher Scientific, Watham, MA, USA). Images were captured using Olympus IX-51 microscope equipped with a DP72 device camera at 20× magnification (Olympus, Waltham, MA, USA). Myocardial fibrosis was quantified as previously described [39]. Briefly, for each section, a set of 10 images was obtained randomly from LV. Scar area was excluded from imaging. Images were analyzed using Image J software (NIH). For each image the percentage of fibrosis was quantified as a ratio of tissue area occupied by collagen (Sirius Red labeled) to the total tissue area within the image. Fibrosis percentage per heart section was calculated as average of collagen percentage per set of images.

### 4.5. RNA Isolation and Quantitative Real-Time RT-PCR

Total RNA was extracted from CD90^+^ cells and left ventricles of Mocetinostat treated and untreated CHF, and sham hearts using PureLink™ RNA Mini Kit (Life Technologies) according to the manufacturer protocol. RNA was then quantified with the Quanti-iT™ RiboGreen^®^ RNA Assay Kit (Life Technologies, Grand Island, NY, USA), and assessed using BioTek Synergy HT Microplate Reader (BioTek, Winooski, VT, USA) (excitation/emission 480 nm/520 nm). Total RNA (200 ng) was reverse transcribed with QuantiTect Reverse Transcription kit (Qiagen, Valencia, CA, USA). Real time RT-PCR was conducted using the Rower SYBR Green Master Mix (Applied Biosystems, Grand Island, NY, USA) on a StepOnePlus Real-time PCR System (Applied Biosystems). Specific primers were synthesized by Life Technologies (Table 1). CYP A was used as a reference gene. Data analysis was performed on StepOne software version 2.1 (Applied Biosystems) using the comparative *C*_t_ (ΔΔ*C*_t_) quantitation method.

**Table 1:** Primers sequences.

Gene	5'-3' Sequence
TGF-β1 F	CGAGGTGACCTGGGCACCATCCATGAC
TGF-β1 R	CTGCTCCACCTTGGGCTTGCGACCCAC
CTGF F	CAGGCTGGAGAAGCAGAGTCGT
CTGF R	CTGGTGCAGCCAGAAAGCTCAA
PDGF F	GGACGCGTAGAACAATCGGG
PDGF R	TGAACGGGTTGCTCGAGGTC
Collagen-1 F	TGCCGTGACCTCAAGATGTG
Collagen-1 R	CACAAGCGTGCTGTAGGTGA
Collagen-3 F	TCCCAGAACATTACATACCACT
Collagen-3 R	GCTATTTCCTTCAGCCTTGA
TIMP-1 F	ATAGTGCTGGCTGTGGGGTGTG
TIMP-1 R	TGATCGCTCTGGTAGCCCTTCTC
Fibronectin F	GATGCCGATCAGAAGTTTGGA
Fibronectin R	TCGTTGGTCGTGCAGATCTC
IL-6 F	CTCTCCGCAAGAGACTTCCA
IL-6 R	GTCTCCTCTCCGGACTTGTG
IL-18 F	CGAACAGCCAACGAATCCCA
IL-18 R	TAGGGTCACAGCCAGTCCTC
Cyp-A F	TATCTGCACTGCCAAGACTGAGTG
Cyp-A R	CTTCTTGCTGGTCTTGCCATTCC

### 4.6. Western Blotting and ELISA

LV tissue was dissected from Mocetinostat treated and untreated CHF, and sham hearts. Dissected tissues were homogenized in lysis buffer (50 mM·Tris-HCl pH 7.5, 150 mM·NaCl, 0.5% NP-40 (Sigma), 0.5% Triton-X (Sigma), 1 mM EDTA (Sigma)), and complete mini protease inhibitor (Roche). Protein concentrations were determined by BCA assay (Thermo Scientific, Omaha, NE, USA). Typically, 40 µg of protein was loaded on 4%–12% Tris-Bis gels (Life Technologies, Grand Island, NY, USA), separated in MOPS running buffer, and transferred to a PVDF membrane (Millipore, Billerica, MA, USA). After blocking with 5% BSA in 1× TBS, membranes were probed with pStat3 Y705, pStat3 S727, Stat3 (1:1000; Cell Signaling Technologies, Danners, MA, USA), HDAC1, HDAC2 (1:1000; Abcam, Cambridge, MA, USA) and GADPH (Sigma-Aldrich, 1:10,000) antibodies over night at 4 °C. Following three washes in TBS, membranes were incubated with HRP-conjugated secondary antibodies (Santa Cruz, Dallas, TX, USA, 1:50,000) for 1 h at room temperature. An ECL (Millipore) system was used for detection of the bands and exposed to X-ray film (Thermo Scientific, Omaha, NE, USA) in a dark room. Densitometry analysis was performed with Alpha Ease FC software (Santa Clara, CA, USA).

IL-6 ELISA (Life Technologies, Grand Island, NY, USA) was performed as instructed in the manufacturer’s manual with conditioned media of CD90+ fibroblasts derived from Mocetinostat treated and untreated CHF and sham hearts.

### 4.7. Statistical Analysis

All data sets are represented as mean ± S.E. Significance (*p* < 0.05) was determined using Student’s *t*-test. Statistical analysis was conducted using Sigma Stat 3.5 software (San Jose, CA, USA).

## Supplementary Materials

Supplementary materials can be found at http://www.mdpi.com/1422-0067/16/05/11482/s1.
