# Supplementary material for: Anti-Fibrotic Effects of Class I HDAC Inhibitor, Mocetinostat Is Associated with IL-6/Stat3 Signaling in Ischemic Heart Failure
Source: Int J Mol Sci. 2015 May 19;16(5):11482–99. doi: 10.3390/ijms160511482 (PMC4463712; doi:10.3390/ijms160511482)
Supplement: Supplementary file 1 [file ijms-16-11482-s001.pdf]

# Supplementary Information

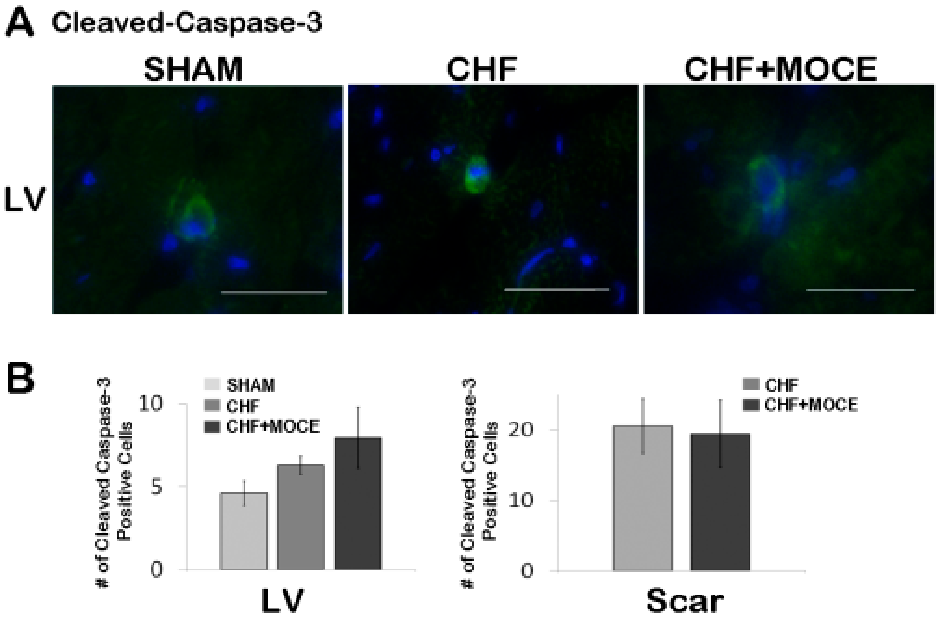


**Figure S1.** Cleaved-Caspase 3 levels were unchanged in Mocetinostat treated animals. Cross section of LV and Scar tissue were stained with Cleaved-Caspase 3 antibody in sham, Mocetinostat treated and untreated CHF hearts. Number of positive cells was counted in LV (**A**) and scar (**B**). Error bars indicate SE. Scale bar 20 µm, *n* = 4. MOCE, Mocetinostat; CHF, congestive heart failure.
